# Supplementary material for: Enhanced nonlinear optomechanics in a coupled-mode photonic crystal device
Source: Nat Commun. 2023 Mar 18;14:1526. doi: 10.1038/s41467-023-37138-z (PMC10024728; doi:10.1038/s41467-023-37138-z)
Supplement: Supplementary file 1 — Supplementary Information [file 41467_2023_37138_MOESM1_ESM.pdf]

Supplementary Information to "Enhanced nonlinear optomechanics in a coupled-mode photonic crystal device"

Roel Burgwal and Ewold Verhagen*
Department of Applied Physics and Eindhoven Hendrik Casimir Institute, Eindhoven University of Technology, P.O. Box 513, 5600 MB Eindhoven, The Netherlands and Center for Nanophotonics, AMOLF, Science Park 104, 1098 XG Amsterdam, The Netherlands
(Dated: February 3, 2023)

SUPPLEMENTARY NOTE 1: EXPERIMENTAL SETUP

Supplementary Fig. 1 shows a schematic of the setup used, where different functional parts have been identified with a coloured background.

The laser lock serves to lock the LO and measurement laser at a fixed frequency offset. This is achieved by creating sidebands at f'_{lock} of the LO laser and measuring the slow beating of one of these sidebands with the measurement laser that is placed close in frequency. This signal is processed by a Red Pitaya digital signal processor, which uses a digital phase-frequency comparator to stabilize the beating to 40 MHz. To set up the correct signal processing on the Red Pitaya, the PyRPL software package was used [1]. The error signal is fed back proportionally to the LO laser laser current and, after integration, to the piezo of the tunable laser. In this way, the LO and measurement laser at locked at a frequency separation of $f_{\text{lock}} = f'_{\text{lock}} + 40$ MHz. By direct measurement it was established that the beating frequency was stabilized to a linewidth much smaller than the mechanical linewidth of ≈ 3 MHz.

The probe preparation unit creates an intensity modulation at 1 MHz of the weak probe laser that allows a lock-in measurement of the reflected power of this laser. The probe laser power was kept low enough such that the probe reflection around resonance could be well described by a Lorentzian, with negligible thermo-optical bistability, with a typical power of 100 nW. When sweeping the probe laser, the laser wavelength is recorded on a wavemeter (Bristol Instruments 871) with repeatability better than 0.1 pm. By fitting the lock-in reflection measurement, the cavity resonance wavelength is determined, from which the detuning Δ can be calculated.

In the detection block, there are two consecutive switches. In the first switch, option 1 leads to detection of transduction, while option 2 is used for detection of the optical properties of the device by measuring probe beam reflection. Such a measurement of optical properties is done before and after each transduction measurement. In the transduction path, switch option A connects to the direct detection configuration, where light is amplified in an EDFA, filtered at the carrier wavelength by a band-pass filter of 0.8 nm FWHM and detected on a fast photodiode (12 GHz bandwidth). In switch path B, the reflected light is mixed with the local oscillator (LO) and captured on a slower, low-noise photodiode. Switch path C is used before and after each heterodyne measurement for the polarisation check described below.

The vacuum chamber that keeps the sample is filled with nitrogen gas to a pressure of 0.25 bar to prevent oxidation or other contamination of the sample. Some sample degradation is, however, still observed. Over the course of weeks after fabrication, the sample optical mode is observed to shift to higher wavelength and the optical linewidth increases. Additionally, a small shift in mechanical resonance frequencies is observed. This is thought to be a sign of the deposition of some contamination on the device while in the vacuum chamber. The sample can be restored to its original state through a brief oxygen plasma clean, after which device optical and mechanical properties are found to return to original values. The sample was plasma cleaned right before measurements reported in this paper. There is an additional round of cleaning between the data in main text Figs. 2 and 3 and main text Figs. 4 and 5. Importantly, the data of main text Figs. 4 and 5 is taken within one day shortly after plasma cleaning.

Finally, the tuning block serves to digitally control the green laser power and to stabilize it. The green light passes through an electronically-controlled variable attenuator (EVOA), after which a part of the laser power is split off and detected on a photodiode. The detector voltage passed through a proportional-integral controller and fed back to the EVOA.

SUPPLEMENTARY NOTE 2: TRACKING RELEVANT SETUP PARAMETERS

To keep constant all relevant parameters between different measurements, we record input power, reflected power and polarisation. Typically, overall reflectivity, defined as PD reflected power divided by PM input power, is about 13%. This encompasses losses in the circulator, fiber tapering region and fiber-to-waveguide coupling efficiency, which are all occurring twice, once in the input path and once in the reflection path. The transmission through the dimple when detached from the sample is 50%. We can estimate a minimum fiber-to-waveguide coupling efficiency of $\sqrt{0.13} \cdot 100\% = 36\%$.

* verhagen@amolf.nl

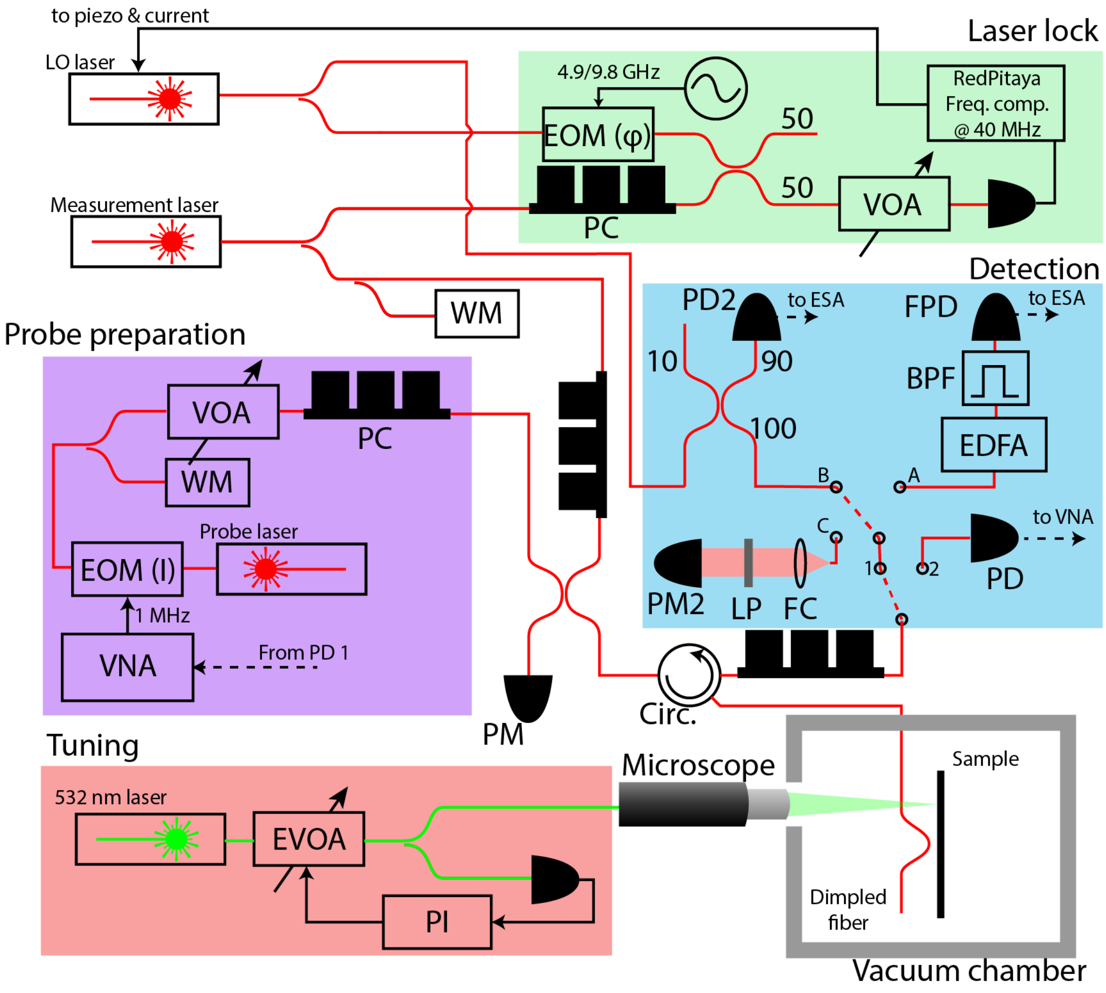

Supplementary Figure 1. **A detailed schematic of the experimental setup.** Different coloured areas group the components by function. Abbreviations used: EOM(ϕ/I): electro-optical phase/intensity modulator, (E)VOA: (electronic) variable optical attenuator, WM: wavemeter, ESA: electronic spectrum analyser, VNA: vector network analyser, PI: proportional-integral controller, PD: photodiode, FPD: fast photodiode, PM: powermeter, PC: polarisation controller, FC: fiber-to-freespace coupler, LP: freespace linear polariser, EFDA: erbium-doped fiber amplifier, BPF: band-pass filter.

To control the polarisation, the combined LO and reflected light are outcoupled into free space and sent through a linear polariser onto a photodetector. The polarisation of both is optimised for transmission through the polariser, ensuring overlap of polarisation of both modes, and measured powers are recorded. This procedure is repeated after each measurement to make sure no significant drift occurred during measurement.

SUPPLEMENTARY NOTE 3: FURTHER COMMENTS ON DATA PROCESSING

The values of laser-cavity detuning Δ are determined from the cavity frequency, found by fitting the probe reflection sweep, and the laser frequency, determined by the wavemeter. It is expected that the fit uncertainty dominates the noise on detuning, while the actual cavity frequency is more stable. Therefore, for heterodyne measurements that sweep only a small range of detunings, we fit a linear function to the cavity frequency versus laser frequency data to extract smoothed values of detuning. The linear slope can capture small cavity frequency shifts due to laser heating.

To isolate optomechanical transduction from the noise background on the heterodyne photodiode, a background subtraction is required. To this end, a noise trace with only the LO is taken before every measurement. The shot noise created by the LO is scaled slightly to match the heterodyne measurement in a frequency region without optomechanical features, after which it is subtracted from the measurement.

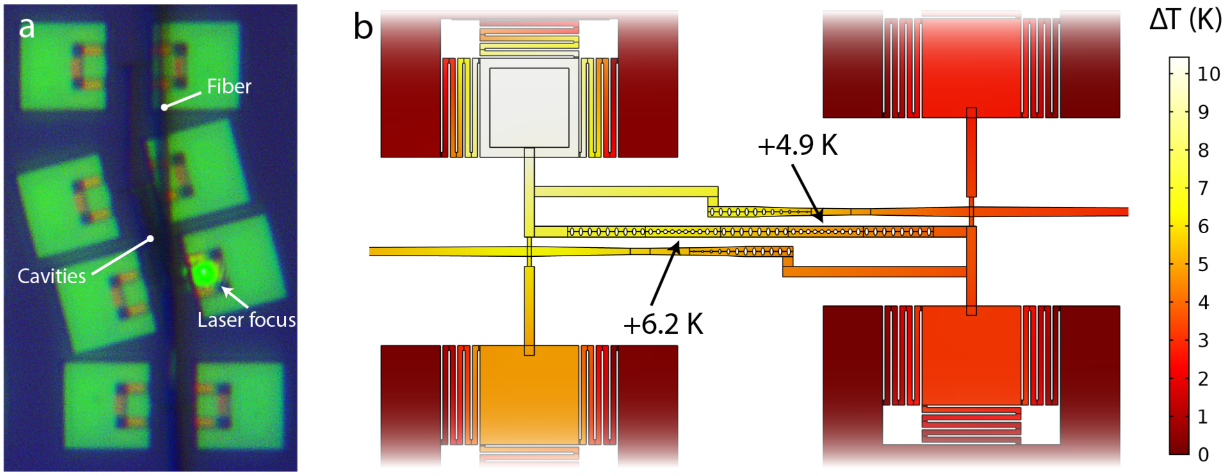

Supplementary Figure 2. **Thermal tuning with a green laser heat source.** (a) An optical microscope image of a fabricated device in the setup, with the 532 nm laser focused on a heating pad and the fiber connected to one of the device waveguides. (b) Simulated temperature increase on the device created by a 20 μW dissipation in one of the heating pads, corresponding to 200 μW tuning laser power. Temperature increase in closest and furthest cavity indicated in figure, giving a relative tuning of 1.3 K.

SUPPLEMENTARY NOTE 4: THERMAL TUNING SIMULATION AND EVALUATION

A 532 nm laser, focused on one of the design heat pads, acts as a heat source for thermal tuning. In Supplementary Fig. 2a, we show an optical microscope image of thermal tuning in the setup. We estimate absorbed power to be roughly 10% of optical power, based on a silicon-air interface reflectivity of 0.3 and an absorption length in silicon for 532 nm of 1.5 μW [2].

The thermal design of the heating pads and tethers was evaluated using COMSOL Multiphysics. In Supplementary Fig. 2b, we show a stationary thermal simulation in which a heat source is placed in one heating pad and the substrate beneath the device acts as a room-temperature heat sink. Because of the thermally isolating properties of the tethers, the closest cavity reaches +6.2 K, with the temperature difference between the cavities at 1.3 K, for a dissipated power of only 20 μW, corresponding to 200 μW laser power. Importantly, for a design without tethers and thus a more direct connection to substrate, a temperature difference between the cavities of only 0.07 K was achieved, meaning that the use of tethers allows us to create a roughly 20 times stronger temperature gradient across the cavities with the same heating power.

Next, the cavity wavelength shift per Kelvin temperature increase can be calculated using a perturbation theory approach [3]:

$$\Delta\lambda = 7.5066 \cdot 10^{-2} \lambda \, n_{\mathrm{Si}}(T) \, \frac{dn_{\mathrm{Si}}}{dT}, \tag{1}$$

where the unitless numerical coefficient characterises the effect of refractive index on wavelength, n_{Si} is the refractive index of silicon and λ is the wavelength. Using silicon material properties from [4], we evaluate that:

$$\Delta\lambda = 73 \text{ pm/K}. \tag{2}$$

Using this value, we can calculate that we expect an absolute wavelength shift of the optical mode closest to the pad of 2.2 pm/μW, and a relative inter-mode shift of 0.47 pm/μW.

From the experimental data, we can determine the tuning coefficient by applying a linear fit to the data of Fig. 2b from the main text. For this, we restrict ourselves to powers below 200 μW, away from the anticrossing, where the mode wavelength scales approximately linear with input power. We find a coefficient of 1.15 pm/μW, a little over half of our simulated coefficient. The discrepancy can possibly be caused by the pad and the laser spot not fully overlapping or by our rough estimation of absorbed power. We can also conclude that at perfect mode tuning for about 400 μW optical power, the device temperature is raised by only 6.3 Kelvin.

SUPPLEMENTARY NOTE 5: SIMULATION OF DEVICE MECHANICAL MODES

After fabrication, the realised dimensions of the device were measured using a scanning electron microscope (SEM), from which it became clear that the beam width was slightly smaller (average −11 nm) and the hole diameter slightly bigger (average +5 nm) than the design. Device simulations were performed in COMSOL Multiphysics using these corrected dimensions. The device phononic crystal with two cavities is simulated, terminated at both ends in a bare waveguide with no holes

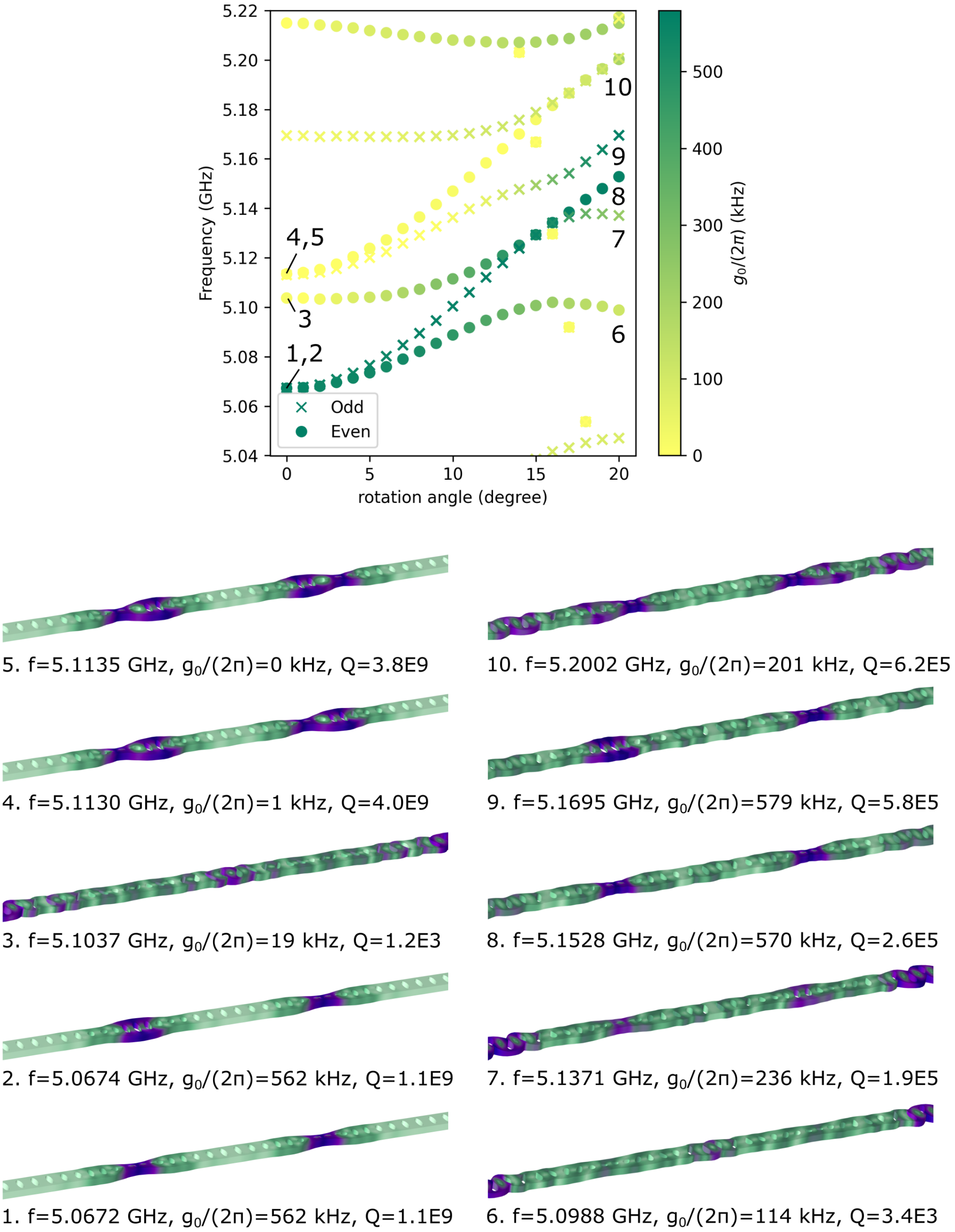

Supplementary Figure 3. **Multiple mechanical modes with optomechanical coupling varying with angle to silicon crystal axis.** The plot displays the eigenmodes of the double cavity nanobeam, with the marker indicating the x-symmetry of the mode (cross is odd, circle is even). The color of the marker indicates the optomechanical coupling strength $g_0/(2\pi)$. Numbers 1-10 indicate specific eigenmodes at $\theta = 0, 20$, of which the mode shape is plotted in the panels below.

which leads into a perfectly matched layer (PML) to simulate leaking into the substrate. The anisotropic mechanical properties of the crystalline silicon mean that relation between stress and strain in the material depends on the direction of the stress and strain with respect to the crystal axis. This is caught in an elasticity matrix [3], which is chosen such that silicon crystal axes $\langle 100 \rangle$ align with the x ,y and z direction of the simulation geometry. The nanobeam geometry is aligned along the x axis and subsequently rotated in plane with an angle θ to simulate the varying mechanical properties of devices fabricated at different angles to the substate crystal axis.

Supplementary Fig. 3 shows how the mechanical eigenmodes of the system change as the angle θ to the crystal axis is varied. At $\theta = 0$, for which the cavities are designed, we see odd and even supermodes of the fundamental cavity modes (panels 1 and 2) and the second-order cavity modes (panels 4 and 5). The mechanical coupling rates J_{M} are 2 MHz and 5 MHz, respectively. Of these two only the fundamental modes couple to the optical mode, with coupling rate $g_0/(2\pi)$ reduced by $\sqrt{2}$ with respect to individual cavities due to the higher effective mass. Between the two pairs of modes lies a mode with odd y-symmetry (panel 3), not confined by the cavities and thus leaking into the environment more easily, although still confined in the phononic crystal.

When we increase θ, mode frequencies start to shift. The non-zero angle breaks the system's y-symmetry and couples modes of different y-symmetry. As can be seen in Supplementary Fig. 3 panels 6-10 for $\theta = 20°$, the new eigenmodes appear to be combinations of the y-even cavity modes and y-odd waveguide mode at $\theta = 0$. As such, they are less confined in the cavities and new eigenmodes are at a larger frequency separation, without affecting the optical properties of the device. As a consequence, the quality factors of these modes are lower. However, optomechanical coupling $g_0/(2\pi)$ is not reduced.

SUPPLEMENTARY NOTE 6: SIMULATION OF DEVICE OPTICAL MODES

Optical simulations of the device with realistic dimensions give a frequency difference $2J_{\mathrm{O}}$ between the two supermodes of 11.3 GHz, significantly larger than the 5.4 GHz observed in experiment. Although SEM imaging tells us the in-plane dimensions of the device, it is much harder to accurately determine variation along the vertical direction. For example, slanted sidewalls (see Supplementary Fig. 4a) would result in an observed device dimension that overestimates the average dimension along the vertical direction. To emulate slanted sidewalls, we simulate our device with a slightly increased hole size and reduced beam width. In Supplementary Fig. 4b, we see that an additional 9 nm, corresponding to a sidewall angle of just $\phi = 4.8°$, could explain the observed optical splitting.

The enhancement demonstrated here relies on a carefully tuned inter-cavity optical coupling J_{O}, and the above simulation suggests that the optical coupling is sensitive to small changes in sidewall angle or hole dimension. Such sensitivity of J_{O} to fabrication imperfection can possibly be avoided by creating optical coupling in a zipper cavity configuration [5], which can be combined with capacitative tuning of the gap for control over J_{O} [6]. Although this will decouple mechanical modes, enhancement of linear and nonlinear transduction is still possible.

SUPPLEMENTARY NOTE 7: ESTIMATING ENHANCEMENT FACTORS FROM COUPLED-MODE MODEL

Starting from our coupled-mode model equations, we apply some simplifications to extract simple expressions for the approximate enhancement of linear and nonlinear transduction in our particular system.

We start with linear transduction, $S^{(1)}_{\mathrm{II}}[\omega]$. We assume tuned cavities ($\Delta_{\mathrm{L}} = \Delta_{\mathrm{R}} = \Delta$), close to optimal detuning $\mathrm{Re}(\Delta) \approx \Omega_m$ and optimal $J_{\mathrm{O}} \approx \Omega/2$. We assume that we excite only the odd optical mode, such that $\bar{a}_{\mathrm{L}} = -\bar{a}_{\mathrm{R}} = 1/\sqrt{2}\bar{a}_{\mathrm{o}}$, the odd mode steady state being $\bar{a}_{\mathrm{o}} = \frac{i}{\sqrt{2}}\frac{\sqrt{\kappa_{\mathrm{ex,L}}}a_{\mathrm{in,L}}}{\Delta - J}$. Finally, we consider only one odd mechanical mode with $g_{\mathrm{R}} = -g_{\mathrm{L}} = g$. Under these conditions, $M_j(\omega_-)$ is the only resonant term and is much larger than other contributions, such that:

$$S^{(1)}_{\mathrm{II}}[\omega] \approx \kappa_{\mathrm{ex,L}} n_{\mathrm{het}} n_{\mathrm{th}} |M(\omega_-)|^2. \tag{3}$$

At this point, it is convenient to set the heterodyne frequency equal to the input frequency, or $\omega_- = 0$, such that $\omega_- = \omega$. $M(\omega)$ can be simplified to

$$M(\omega) \approx \frac{1}{\sqrt{2}} \frac{g\bar{a}_{\mathrm{o}}}{\omega + \Delta + J} \chi(\omega_-), \tag{4}$$

given a combined answer of

$$S^{(1)}_{\mathrm{II,t}}[\omega] \approx \frac{\kappa^2_{\mathrm{ex,L}} g^2 n_{\mathrm{het}} n_{\mathrm{in}} n_{\mathrm{th}}}{4} \left| \frac{\chi(\omega)}{(\Delta - J_{\mathrm{O}})(\omega + \Delta + J)} \right|^2. \tag{5}$$

This transduced signal peaks at $\omega = \Omega$, $\mathrm{Re}(\Delta_{\mathrm{L(R)}}) = \Omega$ and $J_{\mathrm{O}} = \Omega/2$, where it reads

$$\max(S^{(1)}_{\mathrm{II,t}}[\omega]) = \frac{16\kappa^2_{\mathrm{ex,L}} g^2 n_{\mathrm{in}} n_{\mathrm{het}} n_{\mathrm{th}}}{\Gamma\kappa^4}. \tag{6}$$

Similarly, now assuming detuned modes $\Delta_{\mathrm{R}} \gg \Delta_{\mathrm{L}}, J_{\mathrm{O}}, \Omega$, we can simplify $M(\omega)$ to

$$M(\omega) \approx \frac{-g\bar{a}_{\mathrm{L}}}{\omega + \Delta_{\mathrm{L}}} \chi(\omega), \tag{7}$$

giving

$$S^{(1)}_{\mathrm{II,d}}[\omega] \approx \kappa^2_{\mathrm{ex,L}} n_{\mathrm{th}} n_{\mathrm{in}} g^2 \left| \frac{\chi(\omega)}{\Delta_{\mathrm{L}}(\omega + \Delta_{\mathrm{L}})} \right|^2, \tag{8}$$

with

$$\max(S^{(1)}_{\mathrm{II,d}}[\omega]) = \frac{16\kappa^2_{\mathrm{ex,L}} g^2 n_{\mathrm{het}} n_{\mathrm{in}} n_{\mathrm{th}}}{\Gamma\kappa^2\Omega^2}. \tag{9}$$

Together, this gives an enhancement of linear transduction sideband power of

$$\mathcal{E}_{\mathrm{lin}} = \frac{\max(S^{(1)}_{\mathrm{II,t}})}{\max(S^{(1)}_{\mathrm{II,d}})} = \frac{\Omega^2}{\kappa^2}. \tag{10}$$

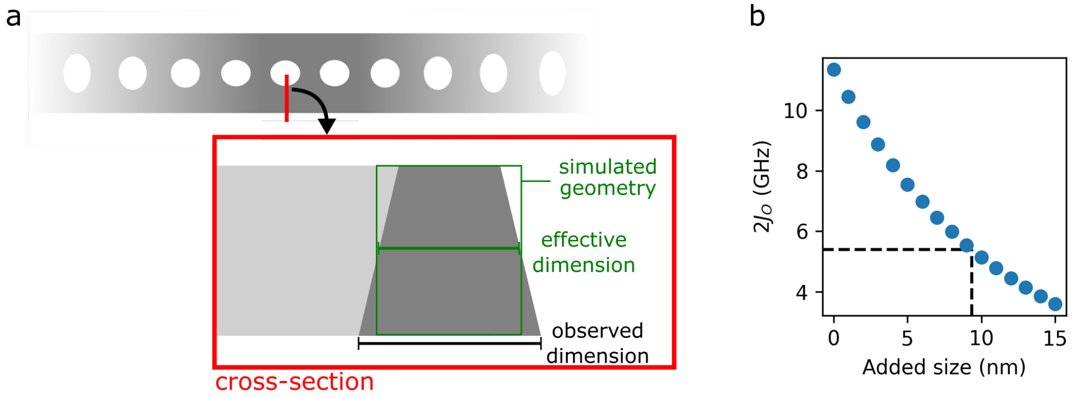

Supplementary Figure 4. **Optical coupling J_O varying due to slanted device sidewalls.** (a) A schematic display of a cross-cut of a fabricated device which has sidewalls at an angle of ϕ from vertical (grey), for which the observed dimension will be an overestimation of device volume. The green outline indicates the geometry that is simulated to estimate the effect of this overestimation. (b) Simulated supermode splitting $2J_\mathrm{O}$ as a function of the added hole size and reduced nanobeam width.

Next, we analyse nonlinear transduction, starting with the tuned system. More precisely, we analyse fluctuations at $2\Omega_j$, assuming Ω_j the eigenfrequency of an odd mechanical mode with $g_{\mathrm{R},j} = -g_{\mathrm{L},j} = g$. Again, we assume Δ and J_O close to optimal, which is $\mathrm{Re}(\Delta) \approx J_\mathrm{O} + \Omega$, $J_\mathrm{O} \approx \Omega/2$, and we assume that we excite the even optical mode. In such case, the expression of $S^{(2)}_{\mathrm{II}}$ is dominated by two terms:

$$S^{(2)}_{\mathrm{II,t}} \approx \frac{\kappa_{\mathrm{ex,L}} n_{\mathrm{het}} n^2_{\mathrm{th}}}{2\pi} \int d\omega' N_{j,j}(\omega_-, \omega')\big(N^*_{j,j}(\omega_-, \omega') + N^*_{j,j}(\omega_-, \omega_- - \omega')\big) \approx \frac{\kappa_{\mathrm{ex,L}} n_{\mathrm{het}} n^2_{\mathrm{th}}}{2\pi} \int d\omega' 2|N_{j,j}(\omega_-, \omega')|^2. \quad (11)$$

$N_{j,j}$ can be simplified with the above assumptions and by assuming only the even optical mode is driven, which in turn entails that $M_{\mathrm{L},j}(\omega) = -M_{\mathrm{R},j}(\omega)$, which means that linear transduction happens only on the odd optical mode. Together, we have

$$S^{(2)}_{\mathrm{II,t}}[\omega] \approx \frac{\kappa_{\mathrm{ex,L}} g^4 n_{\mathrm{het}} n_{\mathrm{cav}} n^2_{\mathrm{th}}}{2\pi} \int d\omega' \left| \frac{\chi_j(\omega')\chi_j(\omega-\omega')}{(\omega + \Delta + J_\mathrm{O})(\omega - \omega' + \Delta - J_\mathrm{O})} \right|^2, \quad (12)$$

where now we consider a constant amount of photons in the cavity n_{cav}. By assuming that the denominator is roughly constant over the frequency range where $\chi(\omega)$ peaks, i.e. the optical properties change much more slowly with frequency than the mechanical properties, we can extract that

$$\max(S^{(2)}_{\mathrm{II,t}}[\omega]) \approx \frac{64\kappa_{\mathrm{ex,L}} g^4 n_{\mathrm{het}} n_{\mathrm{cav}} n^2_{\mathrm{th}}}{\Gamma\kappa^4}. \quad (13)$$

For the detuned case, we have

$$S^{(2)}_{\mathrm{II,d}}[\omega] \approx \frac{\kappa_{\mathrm{ex,L}} g^4 n_{\mathrm{het}} n_{\mathrm{cav}} n^2_{\mathrm{th}}}{\pi} \int d\omega' \left| \frac{\chi_j(\omega')\chi_j(\omega-\omega')}{(\omega + \Delta_\mathrm{L})(\omega - \omega' + \Delta_\mathrm{L})} \right|^2, \quad (14)$$

which gives

$$\max(S^{(2)}_{\mathrm{II,d}}[\omega]) \approx \frac{32\kappa_{\mathrm{ex,L}} g^4 n_{\mathrm{het}} n_{\mathrm{cav}} n^2_{\mathrm{th}}}{\Gamma\kappa^2\Omega^2}. \quad (15)$$

Together, this yields a nonlinear enhancement of

$$\mathcal{E}_{\mathrm{qua}} = \frac{\max(S^{(2)}_{\mathrm{II,t}})}{\max(S^{(2)}_{\mathrm{II,d}})} = 2\frac{\Omega^2}{\kappa^2}. \quad (16)$$

Note that for the equations in the main text, we have set $n_{\mathrm{het}} = 1$ to further simplify expressions.

SUPPLEMENTARY NOTE 8: DETERMINING VACUUM OPTOMECHANICAL COUPLING RATES

The vacuum optomechanical coupling rates were determined using the frequency noise calibration method [7], in which a known phase modulation is applied to the laser as a reference before being sent to the cavity. The applied modulation is detected in direct intensity-modulation detection along with the modulation from optomechanically-transduced thermomechanical motion. By comparing the area of the reference tone (P_{ref}) and the optomechanical tone (P_{OM}) in the electronic power spectral density of the detector, the vacuum coupling rate can be extracted:

$$g_0 = \frac{\phi_0 \Omega_{\mathrm{mod}}}{2\sqrt{n_{\mathrm{th}}}} \sqrt{\frac{P_{\mathrm{OM}}}{P_{\mathrm{ref}}}}, \quad (17)$$

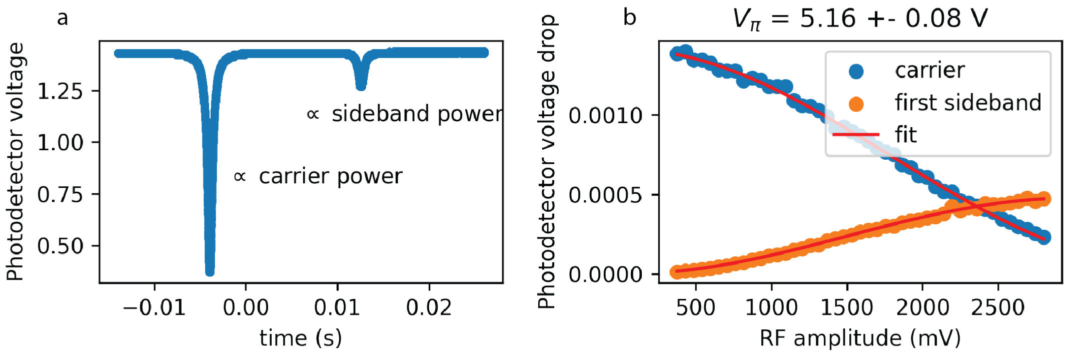

Supplementary Figure 5. **Phase modulator calibration.** (a) An example trace of detected optical power after the fiber-loop cavity taken while sweeping the carrier and sideband over resonance, used to determine optical power of both bands. (b) Extracted carrier and sideband optical powers as function of modulator RF voltage. Data is fitted with Bessel functions to extract V_π.

where ϕ_0 is the amplitude of the applied phase modulation and $\Omega_{\rm mod}$ the modulation frequency. To know the amplitude of phase modulation, the electro-optical phase modulator (EOM) has to be calibrated to retrieve for what voltage amplitude V_π the amplitude of the phase modulation is exactly π, to which end we follow the approach by Schneider *et al.* [8], at the modulation frequency $\Omega_{\rm mod} \approx 4.96$ GHz that is employed in the above procedure. For varying RF voltage amplitude V applied to the modulator, the optical power in the carrier and first generated sidebands are measured by means of a fiber-loop cavity (FLC). The light exiting the EOM is sent through a FLC, after which the total power is detected. When the carrier or the first modulation sideband sweeps across the FLC resonance, the output power dips proportionally to the optical power in the respective band. By slowly modulating the laser wavelength using the laser piezo, the carrier and first sideband power are continuously monitored (see Supplementary Fig. 5a). The carrier and sideband power are fitted with $P_{\rm car}(V) = a\big(J_0(\frac{\pi V}{V_\pi})\big)^2$ and $P_{\rm sb}(V) = a\big(J_1(\frac{\pi V}{V_\pi})\big)^2$, where $J_{0,1}$ are Bessel functions of the first kind and a is a proportionality constant. The data and fit can be seen in Supplementary Fig. 5b, from which we extract $V_\pi = 5.16 \pm 0.08V$. We note that this voltage amplitude is the required signal generator amplitude, already taking into account losses in the electronic cabling to the phase modulator.

The coupling rates $g_{{\rm L(R)},j}$ of mechanical modes $j \in 1, 2, 3$ (denoted by α, β, γ in the main text) to the left or right optical modes are determined by coupling to the device with detuned optical modes via the left and right access waveguide, respectively.

The power measured by the direct detection electronic spectrum analyser of both the OM tones and the reference tone is recorded for various values of laser-cavity detuning. In Supplementary Fig. 6 and 7, we have plotted the measured powers (panels c and d) and extracted vacuum coupling rates (panels a and b) for both the upper (a and c) and lower (b and d) sideband resonance. The datapoints in the green shaded detuning regions were used to determined the coupling rate, as these have the largest signal-to-noise.

Finally, we analyse possible errors on the determination of vacuum coupling rates. Theoretically, the determined coupling rate should remain constant for varying detuning. In Supplementary Figs. 6 and 7, however, we see slight trends in the coupling rates with detuning, and slightly different values for the positive and negative detuning datasets. This last fact can be attributed to the reference tone having less power on the upper sideband resonance (negative detuning) than on the lower sideband resonance (positive detuning), as can be seen from the panels c and d of Supplementary Figs. 6 and 7.

All of these observations can be explained by a slight residual amplitude modulation (RAM) on the incident phase modulation reference tone. We model RAM to predict the error that is imparted on our calculation of vacuum coupling rates. We generate instances of RAM with random amplitude and phase with respect to the phase modulation, calculate the expected reference tone area versus detuning and select such instances as give comparable imbalance between the upper and lower sideband as observed in our experiment. For these instances, the influence on the retrieved vacuum coupling rate was determined by averaging over 1 GHz windows positioned on both upper and lower sideband resonance, analogous to the treatment of experimental data. From this, it was determined that the RAM gives an average relative vacuum coupling rate of 1.01 ± 0.02, i.e. an overestimation of on average 1% with standard deviation of 2%.

We conclude that the vacuum coupling rate should be the average over upper and lower sideband resonance, corrected for the 1% overestimation and with a combined standard deviation due to RAM and V_π uncertainty of 3% ($\sqrt{0.02^2 + (0.08/5.14)^2} = 0.03$).

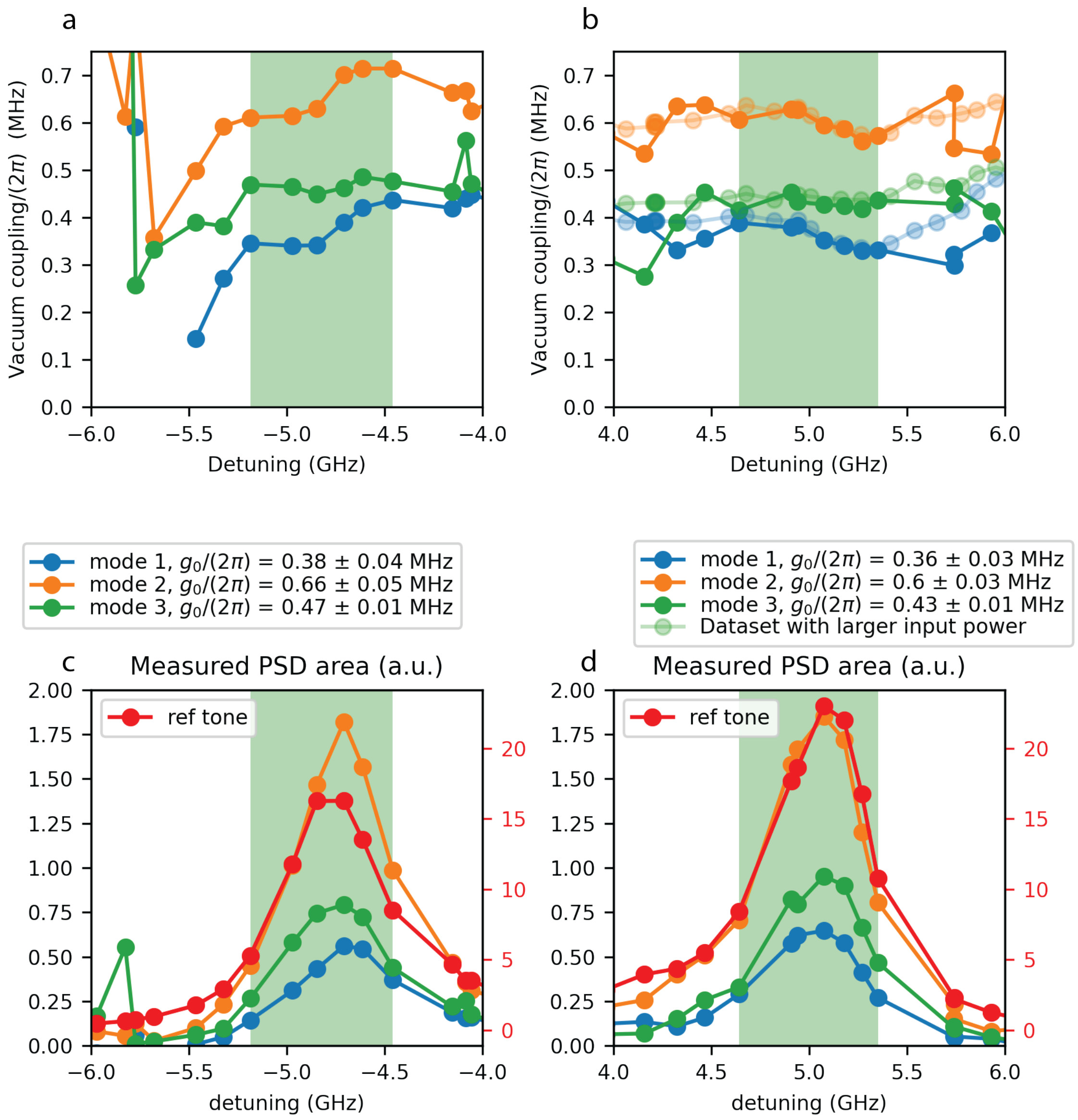

Supplementary Figure 6. Determination of vacuum optomechanical coupling rates to right optical mode. (a) and (b) extracted vacuum coupling of the different mechanical modes to the right optical mode determined around upper (a) and lower (b) sideband resonance. (c) and (d) measured optomechanical and reference tone powers underlying the vacuum coupling rate calculation of (a) and (b). The green shaded area depicts the range of detuning values from which data points are used to determine the average value of vacuum coupling.

SUPPLEMENTARY NOTE 9: TRACKING OF OPTICAL LINEWIDTH AND OUTCOUPLING RATE DURING MEASUREMENTS

During measurements of transduction, relatively large optical input powers are used. It is important to make sure these optical powers do not significantly alter the optical resonance wavelength or linewidth. The optical resonance can be shifted because of unwanted heating due to absorption, while the linewidth can be affected through absorption by free carriers generated by the optical field. To keep track of optical parameters, the optical mode is scanned by a weak probe laser for all values of control laser-cavity detuning (see Supplementary Note 1 for details).

For the measurements of linear transduction as displayed in main text Fig. 4, the measured optical linewidth is plotted in Supplementary Fig. 8a. From the depth of the probe reflection dip on resonance, the outcoupling rate can also be extracted, which is plotted in Supplementary Fig. 8b. The exact optical resonance frequency is used to determine the real laser-cavity detuning, which is used as x-axis. For the tuned device measurements, the two optical supermodes are fitted sep-

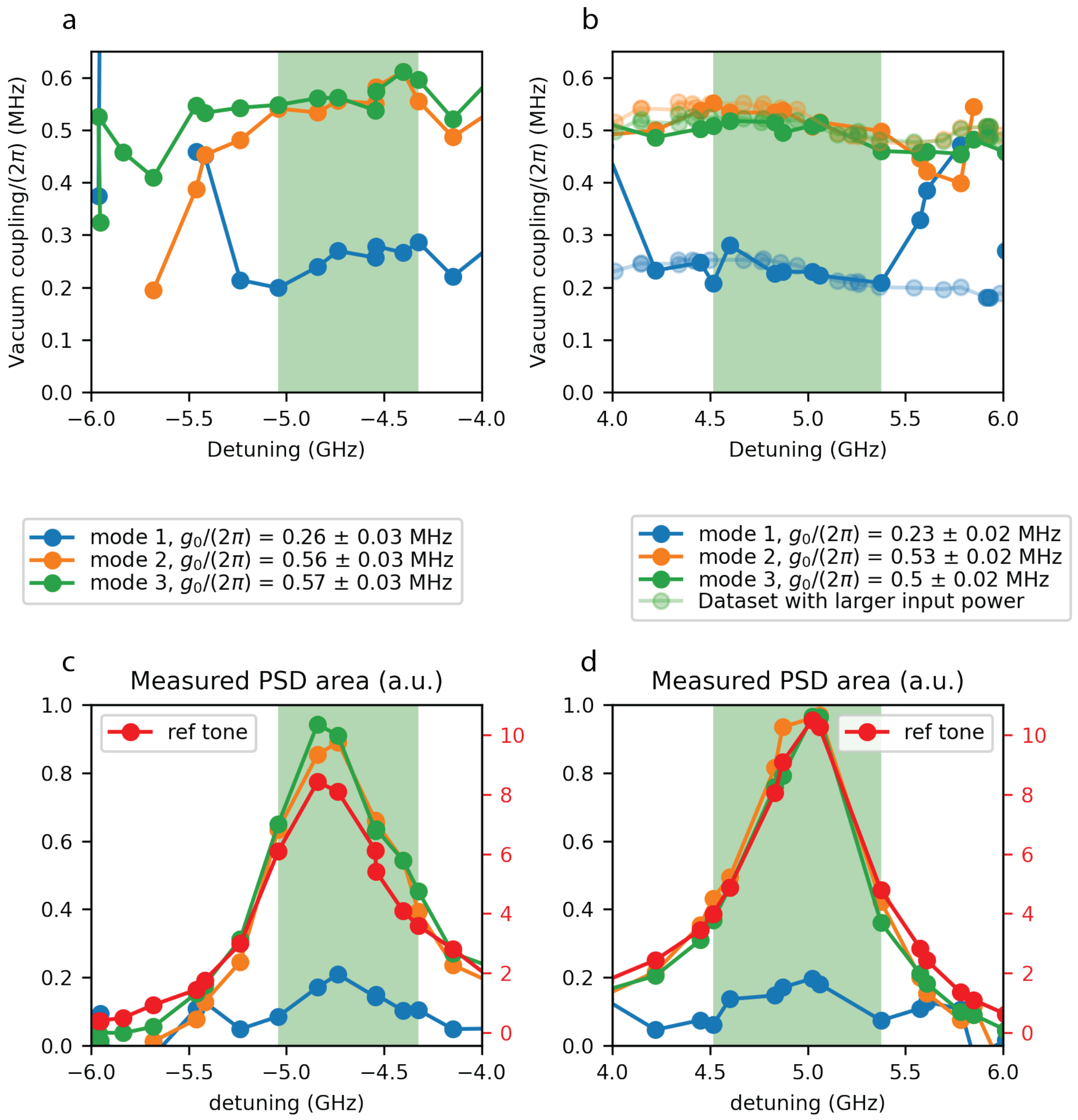

Supplementary Figure 7. Determination of vacuum optomechanical coupling rates to left optical mode. (a) and (b) extracted vacuum coupling of the different mechanical modes to the left optical mode determined around upper (a) and lower (b) sideband resonance. (c) and (d) measured optomechanical and reference tone powers underlying the vacuum coupling rate calculation of (a) and (b). The green shaded area depicts the range of detuning values from which data points are used to determine the average value of vacuum coupling.

arately and linewidth and outcouping rates are extracted. Compared to the detuned device, we expect the effective coupling rate for supermodes to be smaller by a factor 2 due to the delocalised nature of the modes, which is indeed the case for our measured data. Similar measurements are shown in Supplementary Fig. 8c and d for the nonlinear data of main text Fig. 5.

For both linear and nonlinear transduction measurements, this suggests that the optical linewidth is hardly affected by the varying optical powers used. For the largest powers in the optical cavities, as used in the nonlinear measurements, we see a small influence on the effective outcoupling rate, which seems to decrease by about 10-15% moving from linear to nonlinear measurements. The mechanism for this effect is not understood. Such a decrease in outcoupling rate would predict a slightly smaller measured nonlinear transduction than expected.

From this data, we make a rough estimate of the amount with which optical linewidth varies between different transduction measurements. We extract a standard deviation for optical linewidth of 50 MHz and of optical outcoupling rate of 15 MHz.

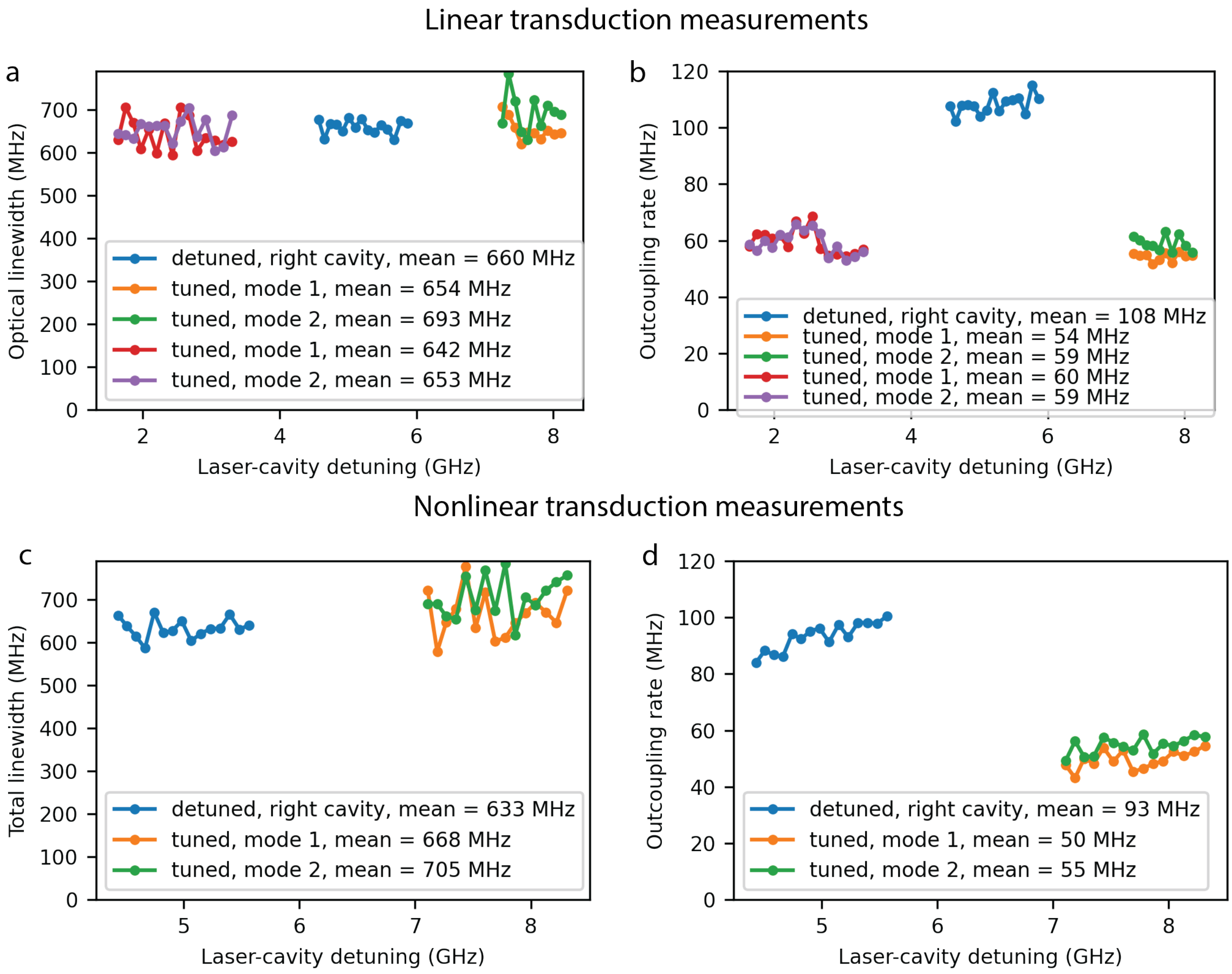

Supplementary Figure 8. **Optical characterisation during measurements of transduction.** (a) Extracted linewidth of the optical mode(s) during measurements of linear transduction. (b) Extracted outcoupling rates κ_{ex} for the linear transduction measurements. (c) Extracted linewidth of the optical mode(s) during nonlinear transduction measurements. (d) Extracted outcoupling rates for nonlinear transduction measurements.

[1] L. Neuhaus, R. Metzdorff, S. Chua, T. Jacqmin and T. Briant, PyRPL (Python Red Pitaya Lockbox) - an open-source software package for FPGA-controlled quantum optics experiments, 2017 European Conference on Lasers and Electro-Optics and European Quantum Electronics Conference (2017).

[2] H. Wang, X. Liu, and Z. M. Zhang, Absorption Coefficients of Crystalline Silicon at Wavelengths from 500 nm to 1000 nm, Int. J. Thermophys. **34**, 213 (2013).

[3] J. Chan, A. H. Safavi-Naeini, J. T. Hill, S. Meenehan, and O. Painter, Optimized optomechanical crystal cavity with acoustic radiation shield, Appl. Phys. Lett. **101**, 081115 (2012).

[4] B. J. Frey, D. B. Leviton, and T. J. Madison, Temperature-dependent refractive index of silicon and germanium, in *Optomechanical Technologies for Astronomy*, Vol. 6273, edited by E. Atad-Ettedgui, J. Antebi, and D. Lemke, International Society for Optics and Photonics (SPIE, 2006) pp. 790–799.

[5] M. Eichenfield, R. Camacho, J. Chan, K. J. Vahala, and O. Painter, A picogram- and nanometre-scale photonic-crystal optomechanical cavity, Nature **459**, 550 (2009).

[6] T. K. Paraïso, M. Kalaee, L. Zang, H. Pfeifer, F. Marquardt, and O. Painter, Position-Squared Coupling in a Tunable Photonic Crystal Optomechanical Cavity, Phys. Rev. X **5**, 041024 (2015).

[7] M. L. Gorodetsky, A. Schliesser, G. Anetsberger, S. Deleglise, and T. J. Kippenberg, Determination of the vacuum optomechanical coupling rate using frequency noise calibration, Opt. Express **18**, 23236 (2010).

[8] K. Schneider, Y. Baumgartner, S. Hönl, P. Welter, H. Hahn, D. J. Wilson, L. Czornomaz, and P. Seidler,

Optomechanics with one-dimensional gallium phosphide photonic crystal cavities, Optica **6**, 577 (2019)
.
